# Isolated and Combined Effect of Age and Gender on Neutrophil–Lymphocyte Ratio in the Hyperglycemic Saudi Population

**DOI:** 10.3390/medicina58081040

**Published:** 2022-08-03

**Authors:** Mohammad A. Alfhili, Jawaher Alsughayyir, Ahmed Basudan, Hazem K. Ghneim, Mourad A. M. Aboul-Soud, Mohammed Marie, Ayed Dera, Mohammed Alfaifi, Ali G. Alkhathami, Zuhier A. Awan, Mohammed R. Algethami, Yazeed A. Al-Sheikh

**Affiliations:** 1 Chair of Medical and Molecular Genetics Research, Department of Clinical Laboratory Sciences, College of Applied Medical Sciences, King Saud University, Riyadh 12372, Saudi Arabia; ahmbasudan@ksu.edu.sa (A.B.); hghneim@ksu.edu.sa (H.K.G.); maboulsoud@ksu.edu.sa (M.A.M.A.-S.); yalsheikh@ksu.edu.sa (Y.A.A.-S.); 2 Department of Clinical Laboratory Sciences, College of Applied Medical Sciences, King Saud University, Riyadh 12372, Saudi Arabia; jalsughayyir@ksu.edu.sa (J.A.); mmarie@ksu.edu.sa (M.M.); 3 Department of Clinical Laboratory Sciences, College of Applied Medical Sciences, King Khalid University, Abha 61421, Saudi Arabia; ayedd@kku.edu.sa (A.D.); mhalfaifi@kku.edu.sa (M.A.); agaithan@kku.edu.sa (A.G.A.); 4 Department of Clinical Biochemistry, Faculty of Medicine, King Abdulaziz University, Jeddah 21589, Saudi Arabia; zawan@kku.edu.sa; 5 Department of Clinical Pathology, Al-Borg Medical Laboratories, Jeddah 23437, Saudi Arabia; 6 Ministry of Health, Jeddah 21176, Saudi Arabia; mralgethami@moh.gov.sa

**Keywords:** diabetes, biomarkers, neutrophil-lymphocyte ratio

## Abstract

Inflammation is pivotal to the pathogenesis of diabetes mellitus (DM), but pathological alterations of the neutrophil–lymphocyte ratio (NLR), an emerging inflammatory index in DM management, remains understudied. The aim of this study is to examine the relationship between NLR and glycemic control in the Saudi population. Gender, age, WBC count, and fasting blood glucose (FBG) were obtained from Al-Borg Medical Laboratories for 14,205 subjects. Means, prevalence, risk measures, and the diagnostic accuracy of elevated NLR and hyperglycemia (HG) were evaluated. Subjects with elevated NLR (>3) had significantly higher FBG (105.10 ± 0.33 vs. 114.0 ± 2.81) and NLR was significantly elevated in impaired fasting glycemia (IFG; 1.21 ± 0.01 vs. 1.25 ± 0.01) and HG (1.21 ± 0.01 vs. 1.39 ± 0.02). Elevations of NLR in HG but not in IFG persisted across all age groups except young males and elderly females. The prevalence of elevated NLR in hyperglycemic subjects was 4.12% compared to 2.16% in subjects with normal FBG. HG was more prevalent in subjects with elevated NLR (17.33% vs. 12.46%) who had a relative risk (RR) of 1.68 (95% CI = 1.38–2.06, *p* < 0.0001) and an odds ratio (OR) of 1.94 (95% CI = 1.48–2.56, *p* < 0.0001) to be hyperglycemic. Nevertheless, NLR failed to discriminate individuals with normal FBG from those with HG based on ROC curve analysis. Pathological fluctuations in NLR may serve as supportive evidence in DM management.

## 1. Introduction

Diabetes mellitus (DM) is a metabolic disease whose pathognomonic manifestation is persistent hyperglycemia (HG) defined by the American Diabetes Association (ADA) as fasting blood glucose (FBG) ≥126 mg/dL [1]. Impaired fasting glycemia (IFG), defined as FBG of 100–125 mg/dL, precedes, and may progress to, overt HG if left untreated [2]. Risk factors for DM include genetic predisposition [3], sedentary lifestyle [4], dietary habits [5], and tobacco smoking [6]. Notably, the interplay between insulin resistance and inflammation contributes to the cytokine and adipokine surge characteristic of DM pathogenesis [7]. Consequently, uncontrolled DM is often complicated by inflammatory conditions, including vasculopathy, retinopathy, nephropathy, and neuropathy [8].

Impaired insulin secretion in type 1 DM (T1DM) to some extent is due to destruction of β-cells by TNF-α, IFN-γ, and IL-1β [9]. In addition to releasing proinflammatory cytokines, macrophages also participate in islet inflammation by creating an oxidative milieu through which cellular injury ensues [10]. Moreover, β-cell death may be instigated by inflammatory infiltrate of T lymphocytes as studied in diabetic mice and humans. Other immune cells; namely, natural killer (NK) cells, NKT cells, and dendritic cells, may similarly modulate T1DM development and progression [11]. In type 2 DM (T2DM), chemotactic white blood cells (WBCs) contribute to vascular injury precipitated by oxidative stress and cytokine release [12]. Although the WBC count has a predictive value for T2DM complications [13], the neutrophil–lymphocyte ratio (NLR), an emerging biomarker, has significant prognostic and stratifying superiority over traditional WBC count in a wide spectrum of inflammatory conditions [14].

NLR represents the absolute counts of circulating neutrophils and lymphocytes, and increased NLR may thus be caused by either elevated neutrophils and/or diminished lymphocytes. As such, NLR serves as a marker of systemic inflammation and disturbed cell-mediated immunity. In theory, physiological stress and pathological conditions in which inflammation or impaired immunity are central to the underlying pathophysiology would result in disrupted NLR. Indeed, investigators have explored the value of NLR in the differential diagnosis of conditions with a similar clinical picture [15,16] and in prognostication of a myriad of diseases [17,18]. Previous reports have found associations between increased NLR and the severity of metabolic syndrome, appendicitis, sepsis, COVID-19, and inflammatory bowel disease, as well as outcome and mortality in tuberculosis, cardiovascular disease, and cancer [19,20,21,22]. In the case of DM, elevated circulating neutrophils may be secondary to inordinate bone marrow production, or a return of resident cells in tissues to the blood [23]. Additionally, interleukins have been shown to cause neutrophilia and lymphopenia during inflammation [24] resulting in elevated NLR.

Because neutrophils utilize glucose as the major source of energy, persistent HG and advanced glycation end (AGE) products drive neutrophil dysfunction in DM. This manifests as impaired defense mechanisms, such as phagocytosis and oxidative burst, which increases susceptibility to infection, as well as augmented NETosis and cytokine release, further exacerbating inflammatory damage and islet cell death in DM [25]. Moreover, HG gives rise to hyperosmotic stress which in turn leads to intracellular Ca^2+^ accumulation, loss of cellular volume, and aberrant neutrophil function [26]. The diabetic microenvironment is also rich in AGE which are potent inducers of reactive oxygen species and upregulate pyroptosis-related mediators in neutrophils [27,28]. Other contributors to neutrophil dysfunction include lipids, infectious and chronic conditions, and aging [25]. Along those lines, a positive association between NLR and glycated hemoglobin A1c (HbA1c) [29] and between NLR and DM complications, including albuminuria and retinopathy, have been established [30,31].

Several lines of evidence point to a promising role of NLR in DM diagnosis and management; nonetheless, population-based studies on the patterns of NLR in HG are severely lacking. Furthermore, the effect of age and gender on the association between NLR and FBG remains understudied, especially in the Saudi population. Thus, the aim of this study is to assess the relationship between NLR and FBG by examining prevalence rates, association estimates, and the diagnostic accuracy of NLR for HG in gender- and age-adjusted analyses.

## 2. Materials and Methods

### 2.1. Study Design, Population, and Data Collection

**Figure 1:**
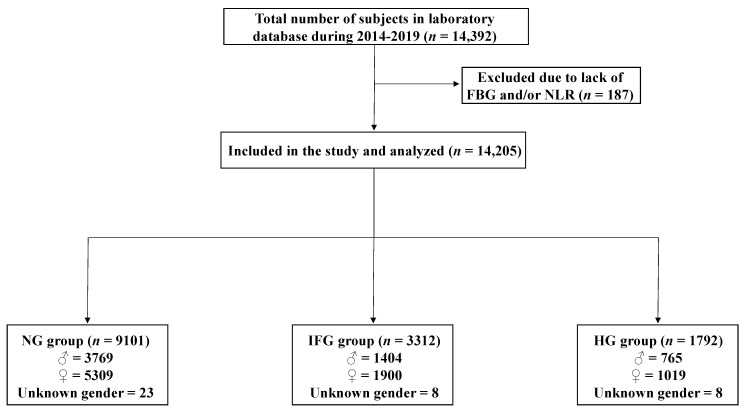
A flow chart of study design.

Following approval of the Biomedical Ethics Unit of Al-Borg Medical Laboratories, Jeddah, Saudi Arabia, gender, age, and laboratory data for 14,205 subjects, collected during 2014–2019, were retrieved from Al-Borg Medical Laboratories database and retrospectively analyzed (Figure 1). Subjects were either walk-ins or referred to the laboratory by a physician. Those with missing information required for a particular analysis were excluded. Subjects were stratified based on gender and age as shown in Table 1.

Blood samples were collected once for each subject in vacutainer tubes coated with sodium fluoride and EDTA for FBG measurement and WBC count, respectively. Subjects were stratified based on FBG cutoffs published by the ADA [1]. Normal glucose (NG) was defined by values <100 mg/dL, IFG by 100–125 mg/dL, and HG by ≥126 mg/dL. NLR ranging from 1 to 3 was considered normal [32].

### 2.2. Statistical Analysis

Data are shown as means ±95% CI (in figures) or SEM (in text) as indicated. Means were compared by either unpaired, two-tailed student’s *t*-test for two groups or one-way ANOVA followed by Tukey’s post-hoc test for three or more groups. Association between FBG and NLR was tested by Pearson correlation and simple linear regression, and risk assessment measures; namely, the relative risk (RR), odds ratio (OR), and absolute risk reduction (ARR), were calculated as permitted by the natural sampling design of the study. All analyses were carried out using GraphPad Prism v9.2.0 (GraphPad Software, Inc., San Diego, CA, USA), and significance was defined by a *p* value of <0.05.

## 3. Results

### 3.1. FBG Is Significantly Increased in Individuals with Elevated NLR

**Figure 2:**
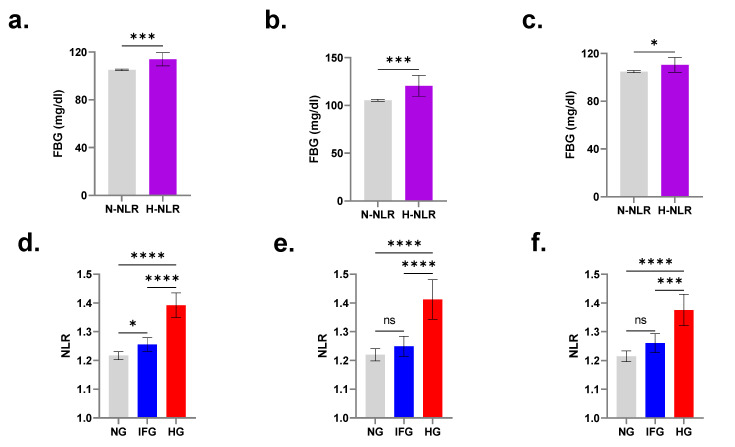
NLR ratios in light of glycemic control. Mean FBG (± 95% CI) in all subjects (**a**), in males (**b**), and in females (**c**). Mean NLR (± 95% CI) of subjects with NG, IFG, and HG in both genders (**d**), in males (**e**), and in females (**f**). * (*p* < 0.05), *** (*p* < 0.001), and **** (*p* < 0.0001).

The total number of participants in our population was 14,205, of whom NG were 9101, IFG were 3312, and HG were 1792. In order to assess FBG levels in light of NLR, subjects of both genders and across all age groups were defined as either of normal NLR (N-NLR) or high NLR (H-NLR). As shown in Figure 1, FBG was consistently significantly increased in the H-NLR group in both genders (Figure 2a; 105.10 ± 0.33 mg/dL vs. 114.0 ± 2.81 mg/dL), in males (Figure 2b; 105.4 ± 0.5 mg/dL vs. 120.4 ± 5.5 mg/dL), and in females (Figure 2c; 104.8 ± 0.43 mg/dL vs. 110.4 ± 3.12 mg/dL).

### 3.2. Subjects with IFG and HG Have Significantly Elevated NLR

Figure 2d shows that those with normal FBG of both genders had an NLR of 1.21 ± 0.01 which significantly increased to 1.25 ± 0.01 in IFG and to 1.39 ± 0.02 in HG. When males and females were considered separately, NLR failed to distinguish between participants with normal FBG and those with IFG. However, it remained significantly elevated in hyperglycemic males (1.22 ± 0.01 vs. 1.41 ± 0.04) and females (1.21 ± 0.01 vs. 1.37 ± 0.03) as shown in Figure 2e,f, respectively.

### 3.3. Age-Controlled Comparisons Displace NLR Significance in IFG

**Figure 3:**
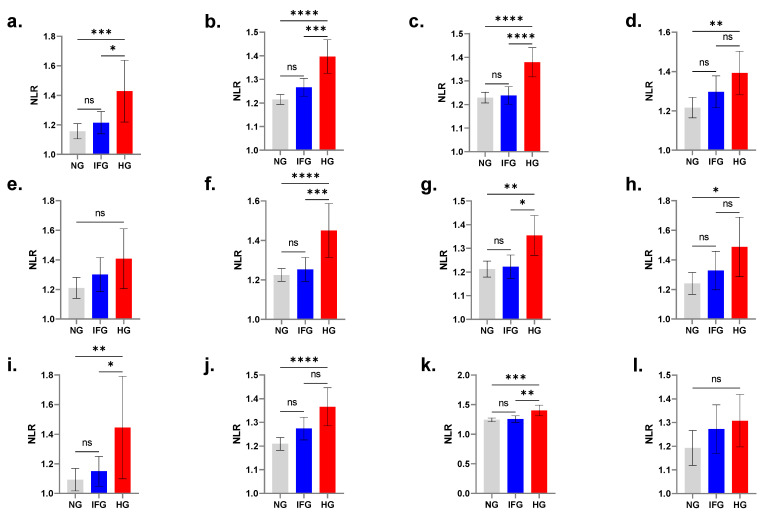
Effect of age and gender on NLR in different glycemic states. Mean NLR (±95% CI) of both genders with NG, IFG, and HG in the young (**a**), young adults (**b**), adults (**c**), and elderlies (**d**). Mean NLR (±95% CI) of males with NG, IFG, and HG in the young (**e**), young adults (**f**), adults (**g**), and elderlies (**h**). Mean NLR (± 95% CI) of females with NG, IFG, and HG in the young (**i**), young adults (**j**), adults (**k**), and elderlies (**l**); ns indicates no significance while * (*p* < 0.05), ** (*p* < 0.01), *** (*p* < 0.001), and **** (*p* < 0.0001).

In both genders, NLR was significantly elevated in hyperglycemic young subjects (Figure 3a; 1.15 ± 0.03 vs. 1.42 ± 0.11), young adults (Figure 3b; 1.21 ± 0.01 vs. 1.39 ± 0.04), adults (Figure 3c; 1.22 ± 0.01 vs. 1.37 ± 0.04), and elderlies (Figure 3d; 1.21 ± 0.03 vs. 1.39 ± 0.06) compared to their normal counterparts.

### 3.4. NLR Is Not Elevated in Hyperglycemic Young Males

Combined age- and gender-wise comparisons revealed that NLR failed to discriminate young males with and without HG as shown if Figure 3e (1.21 ± 0.04 vs. 1.41 ± 0.1). Significantly increased NLR was observed in males with HG across all other age groups including young adults (Figure 3f; 1.22 ± 0.02 vs. 1.45 ± 0.07), adults (Figure 3g; 1.21 ± 0.02 vs. 1.35 ± 0.04), and elderlies (Figure 3h; 1.24 ± 0.04 vs. 1.48 ± 0.10).

### 3.5. NLR Is Not Elevated in Hyperglycemic Elderly Females

Significantly increased NLR was detected in females with HG in the young (Figure 3i; 1.10 ± 0.04 vs. 1.44 ± 0.17), young adult (Figure 3j; 1.21 ± 0.01 vs. 1.36 ± 0.04), and adult (Figure 3k; 1.24 ± 0.02 vs. 1.40 ± 0.05) age groups. However, NLR failed to discriminate elderly females with and without HG, as depicted in Figure 3l (1.19 ± 0.04 vs. 1.31 ± 0.1).

### 3.6. Elevated NLR Is More Prevalent in HG Subjects

The overall prevalence of elevated NLR among all subjects was 3.01%. Table 2 demonstrates that this rate decreased to 2.16% in subjects with normal FBG and increased to 4.71% in IFG and to 4.12% in hyperglycemic participants. A more profound pattern of increase was observed when either gender was considered alone. Likewise, HG was more prevalent in H-NLR compared to N-NLR subjects (17.33% vs. 12.46%) which, again, was consistent in either gender.

### 3.7. Elevated NLR Carries a Greater Risk for HG

As revealed in Table 3, H-NLR males were 2.60 times more likely to have HG and had 3.77 times the chance of having HG compared to males with N-NLR. Similarly, H-NLR females were 2.63 times more likely to have HG and had 3.73 times the chance of having HG compared to females with N-NLR. Additionally, ARR was −4.87% in both genders, −16.58% in males, and −15.59% in females.

### 3.8. Correlation between NLR and FBG

**Figure 4:**
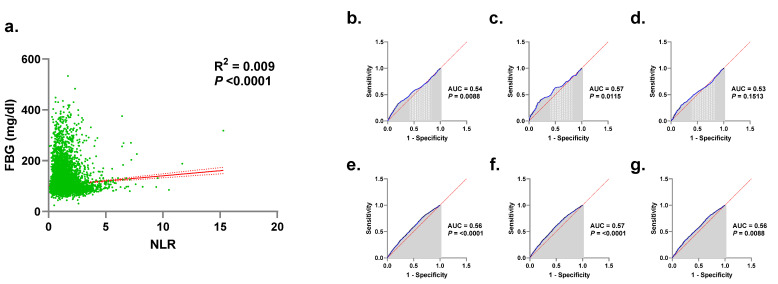
Diagnostic accuracy of NLR: Simple linear regression of the association between NLR and FBG (**a**) and ROC curves of FBG to discriminate N and H-NLR in both genders (**b**), in males (**c**), females (**d**), and of NLR to discriminate NG and HG in both genders (**e**), in males (**f**), females (**g**).

As shown if Figure 4a, our simple linear regression model (R^2^ = 0.009, *p* < 0.0001) indicates that FBG significantly varies with NLR but this variance cannot be solely explained by changes in NLR.

### 3.9. NLR Displays Poor Diagnostic Accuracy for HG

In order to assess the optimal cutoff to maximize the sensitivity and specificity of FBG in discriminating N-NLR and H-NLR, and of NLR in discriminating NG and HG, we analyzed ROC curves for both genders and in males and females. As shown in Figure 4b–d, the area under the curve (AUC) ranged from 0.53 to 0.57 reflective of poor diagnostic accuracy of FBG regardless of gender despite its ability to distinguish between N-NLR and H-NLR in males (Figure 4c) but not in females (Figure 4d). Similarly, as seen in Figure 4e–g, although NLR demonstrated poor accuracy in diagnosing HG (AUC = 0.56–0.57), it nonetheless was able to differ between NG and HG in both genders.

## 4. Discussion

The central finding in our study is that Saudi subjects with HG consistently exhibit significantly higher NLR compared to those with NG, and those with H-NLR have significantly increased FBG levels (Figure 2). IFG does not seem to be associated with H-NLR, which indicates that NLR may not be an early marker of glucose disturbance (Figure 2 and Figure 3). Since the inflammatory state is aggravated in overt DM (i.e., ≥126 mg/dL of FBG), it is not surprising that NLR is not elevated in IFG after controlling for age and gender. In fact, it has been reported that obese Mexicans with IFG have elevated C-reactive protein (CRP) [33], reflective of systemic inflammation. Elevated CRP has also been identified as an independent predictor of pre-diabetic risk in Indians (Jaiswal et al., 2012), and of cardiovascular disease in Bulgarians with IFG [34]. Thus, CRP may be a more sensitive inflammatory marker than NLR, but whether either or both markers are able to inform on disease progression and efficacy of therapeutic intervention remains to be determined in longitudinal studies.

In this report, gender-wise comparisons revealed that NLR failed to distinguish between NG and HG in young males (Figure 3e) and elderly females (Figure 3l). Gender disparity in DM is well established in the literature [35], and, contrary to the majority of the world’s populations, DM is more prevalent in Middle Eastern females than in males [35]. In Turkey, children with T1DM who had lower initial NLR values demonstrated less requirement for insulin than those with higher NLR [36]. Moreover, recent global estimates indicate that DM prevalence peaks earlier in men than in women [37], suggesting sex-dependent differential glycemic control. Along those lines, sex steroids have been postulated as contributing factors behind the diminished susceptibility of females to DM. In particular, endogenous estrogens maintain metabolic homeostasis and loss of aromatase or estrogen receptor activity leads to dysregulated carbohydrate and lipid metabolism [38]. Many reports also indicate that menopausal women administered estrogens had substantially reduced DM incidence [39,40,41], further arguing for the protective effect of steroids against the disease.

It has been estimated that 2.8% of Saudi children and 6.4% of subjects aged 7–18 years have IFG, possibly attributable to an amalgam of risk factors comprised of obesity, inactivity, and excess caloric intake in young Saudis [42]. Although a transient increase in FBG in the young or the increased likelihood of anti-inflammatory medication intake in the elderly cohort cannot be excluded, the gender and age disparity observed in our study warrants further investigation. In our regression model, the variance of FBG levels could not be entirely explained by that in NLR (Figure 4a). A recent study on Turkish diabetic subjects reported that NLR was independently associated with dysregulated glucose control [43], although coexisting inflammatory conditions or complications such as nephropathy may have influenced that association. In patients treated with antidiabetic medications with bona fide anti-inflammatory properties, reduced CRP, inflammatory cytokines, and endothelial dysfunction, along with increased adiponectin were observed. Likewise, anti-inflammatory drugs increase both insulin secretion and sensitivity, and decrease FBG and HbA1c levels [44]. These findings establish the central role of inflammation in DM and validates it as a therapeutic target.

Although the diagnostic value of NLR was found to be poor (Figure 4b–g), previous reports have nonetheless demonstrated the clinical utility of NLR in predicting the development and progression of DM complications. For instance, NLR was an independent risk factor for cerebral hemorrhage [45], a predictor of neuropathy [46], retinopathy [47], and ketoacidosis [48], and correlated positively with carotid atherogenesis [49], nephropathy [43], and lower extremity arterial disease [50]. NLR was similarly associated with gestational DM [51], and with mortality in diabetic COVID-19 patients as compared to nondiabetics [52]. Altogether, it seems that NLR is most appropriate for following up rather than screening or diagnosing DM, but further examination of this is certainly warranted.

The Saudi Abnormal Glucose Metabolism and Diabetes Impact Study (SAUDI-DM), published in 2014, concluded that 22.6% of Saudis had IFG and 11.9% had DM, and that aging beyond 29 and 44 years increases the prevalence of DM to 25.4% and 40.2%, respectively [42]. In our study, the prevalence of HG increased from 12.46% in N-NLR subjects to 17.33% in those with H-NLR, while the prevalence of H-NLR was 2.16% in NG and increased to 4.12% in HG (Table 2). In Turkey, NLR was found to be significantly higher in diabetics but segregation of subjects based on HbA1c displaced this significance [43]. Interestingly, NLR did not significantly differ between Brazilians with normal and high HbA1c nor between those with NG and HG [53]. Large-scale assessment of the inflammatory state in the Saudi population relative to glycemic control remains lacking.

Our study is not without limitations. First, the causality between H-NLR and HG could not be established. Second, our study design does not provide the temporal association between NLR and FBG. Third, data on potential confounding variables, most notably body mass index, lifestyle habits and physical activity, waist circumference, tobacco smoking, supplement or medication intake, and other comorbidities, were unavailable. Nonetheless, our study benefits from the very large sample size which permits generalizability of the results to the Saudi population. In addition, highly efficient data collection and automated laboratory acquisition of multiple variables minimizes analytical variability. In addition, prevalence estimates reflect H-NLR and HG frequency in our population which help inform health planning and policymaking.

## 5. Conclusions

In summary, this report demonstrates that Saudi subjects with HG have elevated NLR in comparison to those with NG and IFG, which was consistent across all age groups, irrespective of gender. The urgent need to identify and validate screening, diagnostic, and prognostic markers of DM is highlighted by recent global estimates indicating that half of DM cases are undiagnosed, and, alarmingly, that 5 million deaths were attributable to DM in 2017. Future studies should thus examine the interplay between HbA1c and NLR, despite no known relation to inflammation, in addition to other traditional and emerging biomarkers such as CRP, CRP/albumin ratio, monocyte–lymphocyte ratio, platelet–lymphocyte ratio, selectins, protectins, and resolvins. Prospective, population-based studies are particularly needed to examine the temporal relation between these markers and the risk, onset, and complications of DM in different clinical contexts.

## Figures and Tables

**Table 1:** Age distribution of study subjects.

Characteristic	Parameter
**Gender**	**Number of Subjects (%)**
*Male*	5938 (41.80)
Young	433 (3.04)
Young adults	2364 (16.64)
Adults	2584 (18.19)
Elderlies	557 (3.92)
*Female*	8228 (57.92)
Young	429 (3.02)
Young adults	4312 (30.35)
Adults	2862 (20.14)
Elderlies	625 (4.39)
*Unknown*	39 (0.28)
**WBC count (×10^6^/mL)**	**Mean (95% CI)**
*Male*	
Young	6.17 (5.98–6.35)
Young adults	6.07 (5.99–6.15)
Adults	6.01 (5.93–6.08)
Elderlies	6.16 (6.00–6.31)
*Female*	
Young	5.92 (5.73–6.12)
Young adults	5.98 (5.92–6.04)
Adults	6.04 (5.97–6.11)
Elderlies	6.02 (5.86–6.17)
**NLR**	**Mean (95% CI)**
*Male*	
Young	1.24 (1.19–1.30)
Young adults	1.25 (1.22–1.28)
Adults	1.23 (1.20–1.26)
Elderlies	1.30 (1.24–1.37)
*Female*	
Young	1.15 (1.08–1.22)
Young adults	1.24 (1.21–1.26)
Adults	1.26 (1.23–1.29)
Elderlies	1.23 (1.18–1.28)

**Table 2:** Prevalence of IFG and HG relative to NLR.

Parameter	NG	IFG	HG
**Both genders**			
N-NLR	97.84	95.29	95.87
H-NLR	2.16	4.71	4.12
**Males**			
N-NLR	98.14	96.08	93.33
H-NLR	1.86	3.92	6.67
**Females**			
N-NLR	97.58	94.74	91.56
H-NLR	2.41	5.26	8.44

NG, normal FBG; IFG, impaired fasting glycemia; HG, hyperglycemia; N-NLR, normal NLR; H-NLR, high NLR.

**Table 3:** Risk assessment of elevated NLR.

	Score	95% CI	*z* Statistic	*p*
**RR**				
**Both genders**				
IFG	1.68	1.50–1.91	8.48	<0.0001
HG	1.68	1.38–2.06	5.15	<0.0001
**Males**				
IFG	1.64	1.34–2.02	4.82	<0.0001
HG	2.60	2.09–3.24	8.56	<0.0001
**Females**				
IFG	1.70	1.46–1.98	6.84	<0.0001
HG	2.63	2.21–3.13	10.92	<0.0001
**OR**				
**Both genders**				
IFG	2.23	1.80–2.77	7.36	<0.0001
HG	1.94	1.48–2.56	4.79	<0.0001
**Males**				
IFG	2.15	1.51–3.08	4.19	<0.0001
HG	3.77	2.61–5.46	7.04	<0.0001
**Females**				
IFG	2.24	1.72–2.93	5.94	<0.0001
HG	3.73	2.81–4.94	9.15	<0.0001

NG, normal FBG; IFG, impaired fasting glycemia; HG, hyperglycemia.

## Data Availability

The data that support the findings of this study are available from Al-Borg Medical Laboratories, Jeddah, Saudi Arabia, but restrictions apply to the availability of these data, which were used under license for the current study, and so are not publicly available. Data are however available from the corresponding author, M.A.A., upon reasonable request and with permission of Al-Borg Medical Laboratories.
